# Astounding Health Benefits of Jamun (*Syzygium cumini*) toward Metabolic Syndrome

**DOI:** 10.3390/molecules27217184

**Published:** 2022-10-24

**Authors:** Maryam Khalid Rizvi, Roshina Rabail, Seemal Munir, Muhammad Inam-Ur-Raheem, Mir Muhammad Nasir Qayyum, Marek Kieliszek, Abdo Hassoun, Rana Muhammad Aadil

**Affiliations:** 1 National Institute of Food Science and Technology, University of Agriculture, Faisalabad 38000, Pakistan; 2 Department of Agriculture and Food Technology, Karakoram International University Gilgit Baltistan, Gilgit 15100, Pakistan; 3 Department of Food Biotechnology and Microbiology, Institute of Food Sciences, Warsaw University of Life Sciences—SGGW, Nowoursynowska 159 C, 02-776 Warsaw, Poland; 4 Univ. Littoral Côte d’Opale, UMRt 1158 BioEcoAgro, USC ANSES, INRAe, Univ. Artois, Univ. Lille, Univ. Picardie Jules Verne, Univ. Liège, Junia , F-62200 Boulogne-sur-Mer, France; 5 Sustainable AgriFoodtech Innovation & Research (SAFIR), F-62000 Arras, France

**Keywords:** Jamun, *Syzygium cumin*, metabolic syndrome, diabetes mellitus, obesity, hyperlipidemia

## Abstract

*Syzygium cumini*, also called Jamun, or black plum, is an excellent source of bioactive components such as flavonoids, polyphenols, antioxidants, iron, and vitamin C. The Jamun tree is a tropical evergreen blooming plant and is an important medicinal plant from the Myrtaceae family that has been used for a long time in Indian and other traditional medicines across the world. Jamun is mainly cultivated in Asian countries such as Pakistan, India, Sri Lanka, and Bangladesh. Since ancient medicine, it has been utilized to treat a variety of diseases and physiological conditions. Currently, it is mostly used as a medication to treat various metabolic issues, including diabetes, hyperlipidemia, hypertension, obesity, etc. Therefore, Jamun could serve a beneficial role against metabolic syndrome (MS). In this work, the latest available scientific literature on Jamun was collected and the clinical trials investigating its effect on diabetes, hypertension, obesity, and hyperlipidemia were analyzed to find out how Jamun could improve the symptoms and biomarkers of MS. Overall, the results of this study found a significant association of Jamun with the prevention and treatment of these biomarkers of MS. In many studies, Jamun showed pharmacological modifications not only in MS but in many other diseases as well. Currently, its utilization as a folk medicine for the treatment of patients with MS is widely acknowledged. Hence, the findings of a large number of clinical studies confirmed the ameliorating effects of Jamun against MS due to its antioxidation, antidiabetic, anti-inflammation anticarcinogenic, and hyperlipidemic effects. More research is still needed to determine and identify the Jamun compounds and to elucidate their mechanisms of action that are responsible for these astounding bioactive properties and health benefits.

## 1. Introduction

Metabolic disorders are serious health issues in today’s world, and the prevalence of metabolic disorders is increasing day by day owing to the adaptation of imbalanced lifestyle patterns, which ultimately has raised the societal health burden of metabolic syndrome (MS) [1]. MS, commonly referred to as insulin resistance syndrome, is a collection of risk factors for type II diabetes mellitus (T2DM) and cardiovascular disease (CVDs). Over a billion individuals are expected to be affected by MS globally. According to the World Health Organization (WHO), it is a medical disorder that is characterized by visceral obesity, insulin resistance, high blood pressure, and abnormal cholesterol levels [2]. MS distribution is geographically diverse, emphasizing the relevance of environmental and lifestyle variables as key causes. The majority of the mechanisms are activated by visceral obesity. However, neurohormonal activation, insulin resistance, and inflammation are important factors in the initiation, progression, and transition of MS to CVD [3].

Diabetes is the most prominent biomarker of MS. It is known as the “third killer” of humanity, roughly affecting 10% of the world’s natives today. It is one of the top 10 causes of death in the world, killing around 1.6 million people each year due to oxidative stress and inflammation caused by hyperglycemia. The hyperglycemia-induced oxidative stress and inflammation are mainly linked with the onset and progression of T2DM. Several studies have found that persistent low-grade inflammation is linked to an elevated risk of T2DM, or this underlying inflammation causes insulin resistance that is linked with symptoms of MS including hyperglycemia [4]. T2DM is a long-term endocrine metabolic condition defined by hyperglycemia and caused by changes in carbohydrate, lipid, and protein metabolism. Recent changes in population lifestyle have led to an increase in prediabetes and now prediabetes is currently treated with dietary restrictions, exercise, and other lifestyle changes. For a better treatment of prediabetes, better medications with an acceptable safety profile are required. In comparison to traditional antidiabetic medicine metformin, one such promising chemical of Jamun (*Syzygium cumini*), has an antihyperglycemic action [5]. 

Hypertension, a biomarker of MS, is the most prevalent cause behind CVDs worldwide, and it is also a major risk factor for the development of other diseases such as MS, diabetes, renal dysfunction, congestive heart failure, coronary artery disease, stroke, etc. [6]. Preeclampsia (PE) is a condition marked by high blood pressure, proteinuria, or edema. This health issue is one of the major causes of mother and child illness and death, with an unknown origin [7]. Hyperlipidemia is characterized by an abnormal rise in the amount of more than one lipoprotein in the circulation, such as triglycerides (TAGs), total cholesterol (TC), low-density lipoprotein cholesterol (LDL-C), or very low-density lipoprotein cholesterol (VLDL-C), while dropped high-density lipoprotein cholesterol (HDL-C) levels [8]. Both hypertension and hyperlipidemia toward the onset of CVDs. According to the 2019 global data, CVDs were responsible for approximately 18.6 million deaths worldwide, with an age-adjusted incidence rate of 6431.6 per 100,000 persons. In contrast, the prevalence of ischemic heart disease (IHD) was 197.2 million persons, with a greater prevalence of 113.7 in males than 83.6 in females, where elevated levels of LDL-C were responsible for 4.4 million global deaths [9].

Obesity is one of the most serious public health issues of our day. Because of the steady but continual growth in its prevalence, it is currently considered a worldwide epidemic. Obesity has an unacceptably high worldwide prevalence, affecting both developed and developing nations, regardless of race, sex, or age [10]. Every year, at least 2.8 million adults die as a result of being overweight or obese. According to the WHO, 650 million persons aged 18 and over were obese in 2016, while around 1.9 billion adults were overweight [11]. Obesity is a serious public health concern that is linked to increased morbidity and death. Obesity increases the risk of a variety of diseases, including arterial hypertension, dyslipidemia, T2DM, coronary heart disease, cerebral vasculopathy, gallbladder lithiasis, arthropathy, ovarian poly-cytosis, and sleep apnea syndrome, as well as various malignancies. The rising difficulty of obesity is its link to major risk factors that elevate the incidence of numerous metabolic disorders, such as CVDs, which cause death worldwide. Obesity-related lipid abnormalities are a key predictor of CVD risk. Obese persons are more likely to acquire dyslipidemia [8]. Unfortunately, the awareness against obesity does not appear to be working: the prevalence has continued to rise in recent years. Obesity may be considered a complex pathology, as well as a persistent low-grade inflammatory condition. In reality, obese persons are at a higher risk of developing comorbidity and morbidity than healthy ones. As a result, the condition worsens, and there is a rise in obesity-related pathologies such as diabetes, cardiovascular disease, and cancer are all examples of chronic diseases. Body mass index (BMI) is the most often used diagnostic metric and is not an appropriate way of measuring body fat. Adopting all viable techniques to prevent obesity, alleviate patient suffering, and lower the societal and therapeutic costs of obesity is critical [12]. 

**Figure 1:**
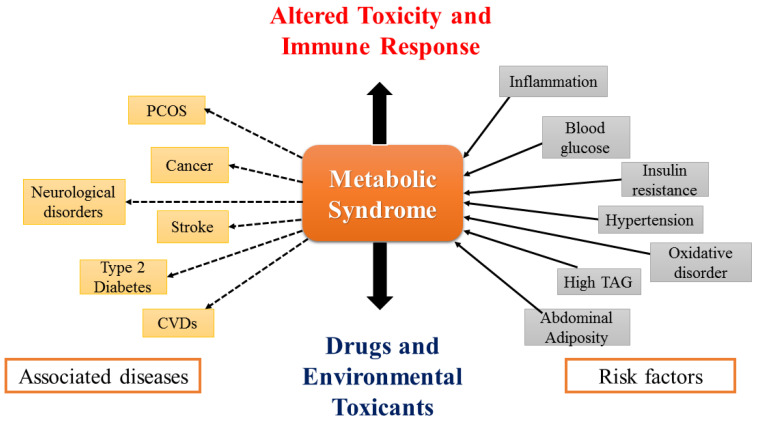
Risk factors of MS and its associated diseases.

MS is a collection of dangerous elements that may act as interrelated variables (elaborated in Figure 1) to increase the likelihood of cancer, T2DM, CVD, and other health issues. Currently, researchers were focused on dietary components that have the potential to prevent numerous chronic illnesses. The inflammation-decreasing effects of anthocyanins and other phenolics are effective at minimizing metabolic alterations and inflammation. The primary issues that need to be addressed are dyslipidemia (high levels of LDL, TG, and low levels of HDL), high blood pressure, obesity, and inefficient glucose metabolism or insulin resistance [3]. In the current review, ethnopharmacological investigations have been focused on the antidiabetic, anti-hypertensive, anti-obesogenic, and anti-hyperlipidaemic properties of Jamun. For this purpose, the last five years’ clinical investigations have been scrutinized using the keywords: “Jamun”, “Java Plum”, “*Syzygium cumini*”, “metabolic syndrome”, “diabetes”, “hypertension”, “obesity”, and “hyperlipidemia”.

## 2. Jamun against Metabolic Syndrome 

Health-protective functional foods are receiving greater attention in the nutrition sector because they support good health while lowering medication costs [9]. Plants have been used to treat and cure a variety of health conditions since ancient times. Ayurvedic medicine is widely used due to its few documented adverse effects and a plethora of benefits [13]. Although ayurvedic medicine, an ancient style of treatment practised in the Indian subcontinent, is thought to be free of side effects, few clinical cases have reported the likelihood of adverse effects associated with the use of ayurvedic medications, for example, consumption of swarnabhasma (gold salt) in liver disease may worsen the condition and ayurvedic medicine containing reserpine in hypertensive man may pose negative health effects [14]. 

Plants contain a variety of free radical scavenging chemicals such as phenolic compounds, terpenoids, nitrogen compounds, as well as vitamins, all of which have strong antioxidant properties [15]. Medicinal herbs are abundant in the plant kingdom and have a wide range of therapeutic uses. The value of such plants or herbs is found in their secondary metabolites, which are non-nutritive yet can have nutraceutical effects in humans against many infectious illnesses and metabolic issues. In contrast to typical diets, nutraceuticals are foods or their components that have both nutritional and pharmacological benefits and are accessible in the form of powder/pill/dietary supplements or food products containing concentrated food derivatives [2]. 

Jamun is a plant that can be used to treat metabolic syndrome-related symptoms all over the world [4]. The Jamun tree is a valuable medicinal plant of the *Myrtaceae* family that has long been utilized in Indian and international traditional medicine. This is an essential medicinal plant used in Pakistani, Indian, Sri Lankan, and Bangladeshi health care systems that have been used to cure a variety of ailments in the past [16] and is an evergreen flowering plant of tropical areas belonging to these countries. Its seeds are also known as “Maghz-e-Jamun or Tukhm-e-Jamun” and have an antihyperlipidemic action in intermediate hyperglycemia. The global output of Jamun is expected to be 13.5 million tons, with India accounting for 15.4% [17]. Jamun is often known as black or java plum [18]. Jamun is a silky smooth and well-developed tree with white branch tips and reddish-brown juvenile shoots that reach a height of 8 to 15 m. The leaves are opposite, glossy and leathery, obovate to oval or obovate-elliptic, 6 to 12 cm long, with a broad and sharp tip [19]. Physical characteristics included the color of its fruit, dark purple, and the color of its seed ranging from white to pink. The Jamun fruit and seed were reported to be 31 and 18.20 mm in length, 28.7 and 11.05 mm in width, and 18.32 and 1.62 g in weight [17]. 

Jamun’s pharmacological activities in the Unani system of medicine include astringent, hemostatic, urinary incontinence, antidiabetic, sexual tonic, and many others. Various portions of the Jamun have been claimed to have antidiabetic, antioxidant, hypolipidemic, ulcer onset prevention, nitric oxide scavenging, free radical scavenging, or radioprotective properties. The medication has been demonstrated to have antidiabetic action both in vivo as well as in vitro [18]. It has astringent, carminative, stomachic, diuretic, antidiabetic, anti-diarrheal, anti-inflammatory, radioprotective, gastroprotective, antioxidant, anti-allergic, anticancer, antibacterial, and cardioprotective effects, among other things. The numerous chemical ingredients present in seeds create glycosides, a trace of pale yellow essential oil, fat, resin, albumin, chlorophyll, an alkaloid-jambosine, gallic acid, 1-galloylglucose, 3-galloylglucose, quercetin, and metals such as zinc, chromium, vanadium, potassium, and sodium [19]. 

Diabetic individuals can gain benefits from its fruit and leaves. The fruit aids in the conversion of carbohydrates to energy and regulates blood sugar levels. Because of its low glycemic index, diabetic patients should consume Jamun during the summer. It alleviates diabetic symptoms such as excessive urination or pushing. The extracts of the leaves, seeds, and bark are very successful in treating diabetes [20]. Intermediate hyperglycemia (prediabetes) is a metabolic disease in which blood glucose levels rise slightly beyond normal but do not rise to the threshold of diabetes. It has been linked to a variety of micro- and macro-vascular problems. There are many Unani medications with antihyperlipidemic properties, and one of them is Jamun [21]. It is a suitable source of iron and ascorbic acid, and it can even help with heart and liver problems. The dried and powdered seed of the Jamun is frequently used in India to manage diabetes [16]. For years, Jamun seed powder has been used as a natural way to maintain a healthy blood sugar level, as well as treat cardiovascular and gastrointestinal problems [22]. Jamun is among the most perishable small fruits, yet it is also the most nutritious. It is mostly employed in the pharmaceutical industry, especially by diabetic patients. It contains many anthocyanins, which have anti-analgesic qualities [23].

## 3. Nutritional Components of Jamun

The chemical composition of Jamun described in Table 1 includes crude fat 1.02 mg (1.18–4.50%), crude protein 3.84 to 7.17 mg (6.3–8.5%), carbohydrate 22.8 g to 31.6 g (41%), crude fiber 7.01 mg (2.64–16.9%), calcium 0.6 mg (0.41%), and phosphorus 0.072 mg (0.17%) [8,17,24,25]. 

## 4. Bioactive Components of Jamun 

Bioactive compounds are the result of an involuntary developmental mechanism that may be caused by a specific requirement, facilitating beneficial physiological processes and promoting preventative mechanisms for mitigating various diseases and disorders through mutualistic or antagonistic interaction [26]. Certain flavonoids and other phenolic compounds from Jamun have been found to exhibit bioactive potentials. The pharmacological properties of Jamun are due to the rich contents of terpenoids, tannins, anthocyanins, flavonoids, and other phenolic compounds are among the chemical ingredients found in them [27]. Anthocyanins, glucosides, ellagic acid, isoquercetin, kaempferol, and myricetin are among the substances found in the Jamun tree. The abundance of bioactive substances in Jamun roots, barks, leaves, trunk, and roots, also including tannins, phenols, lipids, alkaloids, and flavonoids, contributes to an enormous potential source of health-beneficial medicine and nutrition. The active presence of these compounds sustains pharmacological actions such as antioxidant, antibacterial, antidiabetic, central nervous system (CNS) activity, chemoprevention, anti-inflammatory, anti-allergic, and hepatoprotective characteristics in human health and metabolism [26]. Therefore, it has been used in treating kidney problems, diabetes, indigestion, and diarrhea. Hence, it’s antioxidant, anti-inflammatory, anticancer, antidiabetic, antibacterial, antifungal, and radioprotective properties have all been reported. Moreover, Indian cough, cold, allergic asthma, allergic reactions, inflammation, and piles are all treated with Jamun in ayurvedic medicine [27]. 

In the 17th century, the most important phytochemical investigations of the Jamun identified around 35 components from various portions of the plant, including cyanogenic glycosides, tannins, gallitannins, gallic acid, triterpenoids, and essential oil [27]. Table 2. Elaborates on the bioactive constituents identified in various components of the Jamun plant. Jamun mainly contains polyphenols, flavonoids, phenolic, anti-inflammatory, anthocyanins, gallic acids, tannins, phenols, alkaloids, ellagic acid, glycoside, isoquercetin, kaempferol, myricetin, tannins, flavonols, flavone, and vitamins [28]. The leaves of the Jamun the jamblang plant contain several phytochemicals [29], including phytochemicals, flavonoids, and phenolic compounds such as sitosterol, betulinic acid, crategolic acid, quercetin, myricetin, and kaempferol [30]. These flavonoids-, antioxidants-, and polyphenols-rich Jamun leaves possess anti-inflammatory, anticancer, and hypoglycemic potentials [15]. The seeds of Jamun are rich in antioxidants, flavonoids, protein, and calcium [30]. Terpenoids, glycosides, saponins, flavonoids, phenols, and other chemical components are responsible for glucose inhibition. Jamun includes an essential glycoside called Jambolin, which inhibits starch from being converted into sugar and so aids in blood sugar regulation [18]. Anti-inflammatory, neuroprotective, cardioprotective, antioxidant, anti-mutagenic, multidrug resistance and other pharmacological actions have been linked to the bioactive components of Jamun, especially ellagic acid. Ellagic acid has been sold as a dietary supplement with claims of protection against cancer, CVDs, CKD, and metabolic issues [31]. 

## 5. Therapeutic Effects of Jamun 

The oxidative stress through lipid peroxidation contributes not only to the etiology of T2DM but also to diabetic-related vascular problems. These vascular problems such as coronary heart disease, CVD, stroke, neuropathy, retinopathy, nephropathy, CKD, and other long-term issues associated with diabetes are mainly caused by oxidation-induced DNA and protein damage [31]. The leaves of the Jamun contain a number of phytochemicals that are believed to have antihyperglycemic, antihyperlipidemic, and antioxidant properties [29] and thus are beneficial against these maladies. Ultimately, Jamun has long been utilized as a preventative strategy in the treatment of high glucose levels, swelling, ulcers, or diarrhea, and diagnosis investigations have revealed that it has immunotherapeutic, antitumor, and radioprotective characteristics [26]. The pharmacognostic and biophysical properties of the Jamun seed powder have also been used to hinder or halt the components of MS [40]. Therefore, a detailed and interrogative approach to Jamun’s therapeutic potential against various components of MS has been discussed here.

### 5.1. Antidiabetic Effects of Jamun

Several scientific researches has been established the foundations for great antidiabetic therapeutic potential. The therapeutic potential, when evaluated via an experimental design in a rat model, resulted in the glycemic alterations of the rat models over 8 weeks. Streptozotocin significantly increased blood glucose levels in rats. Jamun dosages of 100 mg/kg and 200 mg/kg minimized blood glucose levels. Jamun 200 mg/kg, both alone and in combination with metformin, resulted in a substantial decrease in HbA1c levels at the end of the eighth week when compared to their baseline values [36]. Similarly, to assess the lipidemic and antidiabetic action of herbal medication and its single ingredient (Jamun seed), rats were divided into four groups: control, glibenclamide, petroleum ether extract of Jamun seed powder (PESE), and herbal medicine (HM-01). On streptozotocin-induced T2DM model rats, the antidiabetic and anti-lipidemic effect of this drug and seed extract was investigated. Serum glucose and serum lipid profile were the parameters tested. Fasting blood glucose (FBG) levels were significantly reduced after 22 days of taking HM-01 and PESE orally [41]. 

The hypoglycemic potential of Jamun extracts was similarly assessed in another study by using male Sprague Dawley rats. For 60 days, ethanolic extracts of Jamun fruit and seeds were fed to hyperglycemic/diabetic rats on a regular or high sucrose diet. To assess the hypoglycemic impact of Jamun extracts, insulin and blood glucose levels were measured at periodic intervals. Both seed and fruit extract dramatically lower blood sugar levels as well as control insulin levels in hyperglycemic rats. Jamun fruit extract reduced blood sugar levels by 12.29% and 5.35% in hyperglycemic normal and normal rats, respectively, while improving sugar levels by 6.19% and 2.82%. In normal and hyperglycemic rats, Jamun seed extract lowered sugar levels by 7.04% and 14.36%, respectively, or exhibited 7.24% and 3.56% raising insulin levels [29]. A human trial was also conducted where a 38-year-old male patient with a T2DM clinical history took 32 (20/12) insulin units each day. Taking into account the Dosha and Dusya, a judicial combination of four fundamental ayurvedic drugs, Gudmar, Jamun, Nagarmotha, and Sudarshan, was recommended to be taken orally in the morning and evening with lukewarm water. The HbA1c was 11.1% at the start. After 12 weeks of following the suggested ayurvedic medication coupled with insulin, the HbA1c level dropped to 5.6%. There was no need for further insulin or oral hypoglycemic agent medicines after the ayurvedic therapy. The HbA1c was constantly evaluated, and it has returned to the normal range, resulting in an enhanced quality of life. Even after discontinuing insulin after 12 weeks of initial ayurvedic therapy, the suggested combination of four medications maintained normal blood sugar levels in the T2DM instance [42]. 

**Figure 2:**
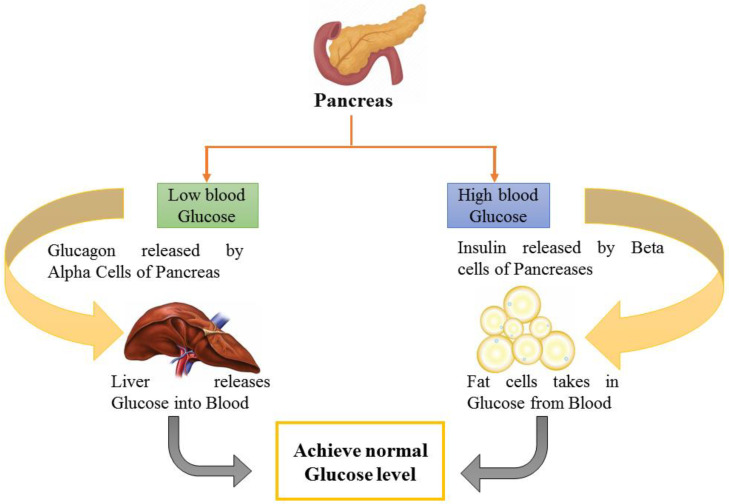
Blood glucose maintenance as facilitated by Jamun.

Another study indicated lowered fasting blood glucose (FBG) levels by 46.67–52.67%, which is comparable to the conventional medication glibenclamide [40]. In another study on streptozotocin-induced T2DM model rats, the antidiabetic and anti-lipidemic effect of this drug and Jamun seed extract was investigated. Chemical analysis revealed that carbs and steroids were present in both samples. Only one herbal medication sample included alkaloids. The arsenic concentration was determined to be 0.05 ppm, which was less than the tolerance threshold. Serum glucose and serum lipid profile were the parameters tested. FBG levels were significantly reduced after 22 days of taking herbal medicine and petroleum ether extract of Jamun orally [41]. Another research was designed to achieve the goal of exploring how a hydro-ethanolic seed extract of Jamun can influence T2DM in a rat model. For 21 days, diabetic rats were administered the 100, 200, or 400 mg/kg ethanolic extract of Jamun. Before and after treatment, the serum levels of insulin, FBG, and lipids were assessed in the various treatment groups. Insulin resistance was tested using a homeostasis model assessment of insulin resistance (HOMA IR), and beta cell function was measured using a homeostasis model assessment of beta cell function. Ethanolic extract of Jamun at dosages of 100 and 200 mg/kg in Wistar rats exhibited statistically significant antidiabetic activity by improving pancreatic body weight (BW) beta cell function and lowering insulin resistance [43]. Such beta cell amelioration and blood glucose maintenance by the consumption of Jamun extract have been depicted in Figure 2, which elaborates on the pathway responsible for glucose maintenance by Jamun consumption.

### 5.2. Anti-Hyperlipidaemic Effects of Jamun

Hyperlipidemia is one of the major health disorders in the current era, which is affecting the young generation in the same proportion as the old ones. Jamun seeds and leaves provide a protection against hyperlipidemia. The group of scientists investigated it for over 30 days, and 20 male albino rats were employed. The animals were separated into four groups, with the first group representing the control group, which was fed a basal diet, and the second group, the hyperlipidemia group, was given a high-fat diet (HFD). Next, the third and fourth groups were fed HFD supplemented with Jamun seeds and leaves, where group number three was given seeds, and group number four was given leaves. Blood samples were obtained at the end of the study to analyze the lipid profile, liver functions, and kidney functions such as urea nitrogen and creatinine. The HFD group of rats was thought to be a key risk factor for hyperlipidemia illness. Jamun seeds and leaves were shown to be the most effective in lowering TC, TAGs, and LDL-C. Furthermore, serum glutamic oxaloacetic transaminase (SGOT), serum glutamic pyruvic transaminase (SGPT), and alkaline phosphatase (ALP) activity were substantially lower in the negative control group than in the positive control group. It is possible to infer that the Jamun leaves and seeds under investigation were effective in the treatment of hyperlipidemia [30]. The anti-lipidemic effect of this Jamun seed extract was investigated in streptozotocin-induced T2DM model rats. The HM-01 and PESE treatment groups reduced TC levels by 25% and 23%, respectively; TAGs levels by 24% and 28%; LDL-C levels by 34% and 35%, respectively; and increased HDL-C by 14% and 22%. In another study, T2DM rat models were fed the herbal medication and its ingredient, Jamun seed powder, which has anti-diabetic characteristics [41].

Another research was conducted on newborn Wistar rats who were administered with either saline as CTRL group or L-monosodium glutamate (MSG) (4 mg/g BW). At 90 days of age, CTRL and some MSG rats were given saline, while others were given 500 mg/kg/day hydroethanolic extract of Jamun leaf for 30 days. Daily vaginal washes were used to identify the estrous cycle. Finally, the animals were slaughtered during their estrous period, blood was drawn to measure sex hormones, and organs were taken for weight and histological analysis. If we compare it with MSG, it controls dyslipidemia. However, it did not show any effects in restoring oligociclicity [44]. Similarly, another study investigated Jamun’s combined antidiabetic and anti-hyperlipidaemic effect on 99 people with poor glycemic control and T2DM, where 50 of them were given 10 g of Jamun seed powder supplementation twice daily right before meals, along with oral hypoglycemic medications as before, and 49 receiving placebos with oral hypoglycemic drugs as previously. All of the patients were monitored for a total of 90 days. The supplements of Jamun seed powder helped improve sugar levels and dyslipidemia. In rural areas, Its low cost may aid the improvement of biochemical parameters in diabetic patients [45]. In another study, Prediabetic patients were randomly allocated to one of two groups: Group A received 4.5 g powder/day of Jamun seed powder in capsule form, whereas Group B received placebo capsules. The lipid profile was evaluated at the start and end of the treatment on the 84th day. The prediabetic study participants’ lipid profiles had dramatically improved, with a particularly notable improvement in TC level [21].

In an investigation of nine weeks, mice were randomly assigned to one of five groups and fed a variety of diets. Group 1 obtained a normal diet (ND), Group 2 received an HFD, Group 3 received an HFD Plus 10% weight-based freeze-dried duhat/Jamun (FDD) powder, Group 4 received an HFD + 20% weight-based FDD powder, and Group 5 received an HFD + 30% weight-based FDD powder. The in vivo investigation found a 50% reduction in blood TG with 30% FDD supplementation and a 30% reduction with 20% FDD supplementation. In addition, a considerable rise of up to 45% in HDL-C was seen in the 30% FDD-supplemented group. Meanwhile, the blood levels of TC in mice were not dramatically altered. The nutritional, as well as bioactive components present in Philippine duhat (Jamun), may be responsible for the improved lipid profile [8].

### 5.3. Anti-Obesity and Anti-Hypertensive Effects of Jamun 

**Figure 3:**
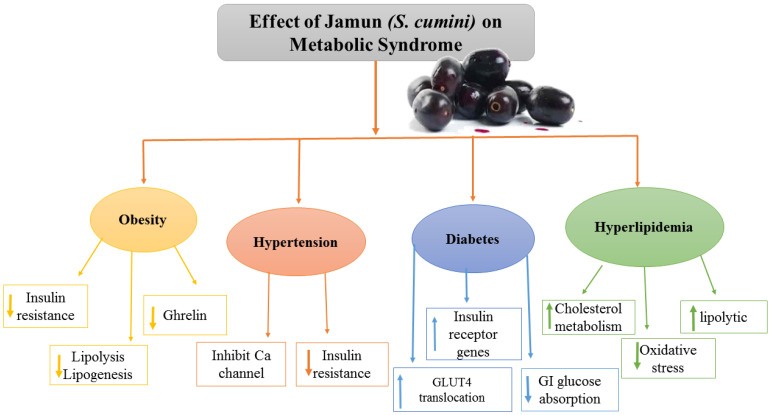
Effect of Jamun on metabolic syndrome.

Jamun helped in BW reduction by attaining the BW of diabetic rats to 18.20–20.41%, as compared to 22.95% in the standard medicine group [40]. If a standardized extract from Jamun a commonly eaten tropical fruit, may reduce obesity and change the gut microbial population in mice fed an HFD. Mice were fed a standard diet (SD) or an HFD with or without Jamun fruit extract (JFE; 100 mg/kg/day) orally for 8 weeks. JFE supplementation significantly decreased diet-induced significant obesity, insulin resistance, and hepatic steatosis. JFE supplementation also repaired HFD-induced gut dysbiosis by restoring the Firmicutes Bacteroidetes ratio, the relative abundance of certain taxa, and the proportion of intestinal short-chain fatty acids. These positive findings suggest a link between gut microbiota regulation and JFE administration metabolism improvement, and they encourage the use and future exploration of Jamun fruit as a dietary intervention method for the prevention of obesity and associated metabolic diseases [46]. Similarly in a study, 20 male Wistar rats were placed into four groups, each with five rats: one was negative control, normal control, and two treatment groups received ethanol extract of Jamun puree at dosages of 100 mg/g and 200 mg/g BW, accordingly. Restraint stress was instilled in the rats by placing them in restraint rats for 30 min each day for seven days. Before and after the therapy, blood pressure was taken, and malondialdehyde (MDA) levels were then measured. A one-way ANOVA was used to evaluate the data. The results revealed that inducing restraint stress considerably elevated the rats’ blood pressure. In chronic stress rats, the injection of an ethanol extract of Jamun pulp considerably reduced the blood pressure rise. Moreover, the levels of MDA in the therapy groups were considerably lower than those in the negative control, demonstrating that an ethanol extract of Jamun pulp might prevent MDA levels from rising. Chronic restraint stress rats blood pressure and MDA levels can be reduced by administering an ethanol extract of Jamun pulp [34]. Figure 3 outlines the overall Jamun’s contributory effects on MS as indicated in the literature. 

### 5.4. Antioxidative and Anti-Inflammatory Effects of Jamun

The seed extract of Jamun is high in phenolic compounds. In alloxan-induced diabetic rats, the extract demonstrated considerable radical DPPH scavenging action and FBS-reducing capability [40]. The scientist investigated the antioxidant, cytoprotective, and thrombolytic capabilities of Jamun extract by using the DPPH scavenging test, the XTT assay, prothrombin, and activated partial thromboplastin time, in that order. Jamun extract was tested for anti-inflammatory and analgesic properties in rabbits using the Carrageenan-induced paw edema and writhing techniques, respectively. Jamun extract had significant quantities of total phenolic (369.75 ± 17.9 mg GAE/g) and flavonoid (75.8 ± 5.3 mg RE/g) content, according to phytochemical analyses. The DPPH experiment revealed a stronger antioxidative potential (IC-50 value of 133 μg/mL), which was comparable to that of ascorbic acid IC-50 (122.4 μg/mL). Furthermore, the Jamun extract exhibited dose-dependent cytoprotection against H_2_O_2_-treated bone marrow mesenchymal stem cells. Jamun possesses anticoagulant activity as well, with a prothrombin time of 28.3 ± 1.8 s compared to 15.8 ± 0.2 s for the control at *p* 0.05. The leaf extract also showed an analgesic effect in rabbits, as evidenced by a reduction in writhing (12.2 ± 1.7 control vs. 3.7 ± 0.6 treated), as well as anti-inflammatory activity in rabbit paw, with a 64.1 ± 2.4% protection against inflammation [15].

In vitro anti-inflammatory efficacy of Jamun fruit extracts were tested utilizing membrane stability, bovine serum albumin denaturation, and egg albumin denaturation assays. Formaldehyde, carrageenan, and prostaglandin E2 (PGE2) produced edema mice models were used to assess in vivo anti-inflammatory activity. Active extracts were fractionated using High-Pressure Liquid Chromatography (HPLC), and the bioactive compounds that were responsible for the anti-inflammatory activity were then identified using liquid chromatography-electrospray ionization/multi-stage mass spectrometry (LC-ESI-MS/MS) analysis. The extract of Jamun fruit includes a high concentration of bioactive chemicals, which should be investigated further as anti-inflammatory medication leads [47]. Similarly, hydroethanolic extract, ethyl acetate, and hydro-methanolic fractions from the leaves of Jamun were tested for antioxidant activity and hypoglycemic effect in rats. All experiments were performed in three replicates. The fractions had no significant effect on blood glucose regulation in non-diabetic mice. The current study concludes that the phenolic chemicals in the plant have antioxidant and hypoglycemic properties [48]. To gauge antioxidant activity, the radical scavenging activity of DPPH was measured. Alloxan-induced (150 mg/kg) diabetic rats were given Jamun seed ethanolic extract at dosages of 200 mg/kg and 400 mg/kg BW for three weeks. The extract includes phenolic components, flavonoids, glycosides, alkaloids, tannins, and saponins, according to phytochemical profiling. The extract contained TPC 177.33 mg GAE/g and showed a considerable percentage inhibition when compared to conventional vitamin C [40]. Various clinical studies performed to investigate the therapeutic potential of different Jamun components against multiple biomarkers of MS, such as obesity, diabetes mellitis, hypertension, and hyperlipidemia, are presented in Table 3. 

## 6. Conclusions

The peel, pulp, and seed of the Jamun are all significant sources of antioxidants, polyphenols, flavonoids, minerals, vitamins, and phytochemicals. Several studies show Jamun’s pharmacological association with metabolic issues. These valuable natural products are widely utilized as folk medicine in various parts of the world for disease treatment. Results from various studies included in this review revealed significant beneficial effects of the Jamun plant, especially the fruit and seeds. The detailed investigation of the nutritional and medicinal properties of Jamun against T2DM, CVDs, hypertension, hyperlipidemia, and obesity indicated several positive health benefits, which, when combined, could increase health protectiveness against MS. Further research on nutrigenetic and nutrigenomic perspectives of Jamun are required to aid the medical sciences and enhance natural ways of halting ailments. Jamun is still an underutilized and underexploited plant. Therefore, its agricultural importance, along with the awareness of its health advantages, is mandatory for increasing its regular consumption. Therefore, it is important to promote wider agricultural and industrial development of this medicinal plant, along with launching campaigns that aim to increase awareness of its health advantages. Innovative ideas on its regular consumption are still awaited, which may enhance the intake of several significant bioactive compounds from Jamun to fight against MS. 

## Figures and Tables

**Table 1:** The nutrition composition of Jamun.

Nutrient	Composition			
Crude fat	1.02 mg	1.18%	-	4.50 ± 0.21%
Crude protein	3.84 mg	6.3–8.5%	4.72–7.17 mg	7.10 ± 0.20%
Carbohydrate	31.62 mg	41%	22.8–29.4 g	-
Crude fiber	7.01 mg	16.9%	3.05	2.64 ± 0.06%
Vitamin A	3 IU/100 g	-	-	-
Vitamin B3	0.09 mg/100 g	-	-	-
Vitamin C	0.21 mg/100 g	-	-	-
Iron	0.140 mg	-	-	-
Calcium	0.651 mg	0.41%	-	-
Magnesium	0.010 mg	-	-	-
Phosphorus	0.072 mg	0.17%	-	-
Potassium	16.07 mg	-	-	-
Zinc	0.009 mg/100 g	-	-	-
References	[17]	[24]	[25]	[8]

**Table 2:** Main components and the bioactive composition of Jamun.

Main Components	Bioactive Composition	References
Jamun fruit	Polyphenols and flavonoids, a rich source of polyphenols anthocyanins, ellagic acid, glycoside, isoquercetin, kaempferol, and myricetin	[32]
	Because of its high anthocyanin content, it is a suitable source of antioxidants Phenolics, alkaloids, tannins, flavonols, tannins, phenols, alkaloids, saponins, and flavone	[33]
	Antioxidants, vitamins, tannin, anthocyanins, flavonoids, tannins, phenols, alkaloids, saponins, ellagic acid, glucoside, isoquercetin, anthocyanins, kaemferol, and myricetin are all components Gallic acid, terpenoids, and alkaloids are all found in this plant	[34]
Jamun leaves	Antidiabetic, polyphenols, flavonoids, terpenoids, saponins, and glycosides	[23]
Jamun leaves extract	Anti-inflammatory, antioxidant, anticancer and hypoglycemic agents, polyphenols, and flavonoids	[35]
Jamun leaf	Sitosterol, betulinic acid, crategolic acid, quercetin, myricetin, methyl gallate, and kaempferol are examples of flavonoids, terpenoids, and phenolics	[36]
Jamun seed	Antioxidant activity and rich source of protein and calcium, flavonoid, antioxidant phytochemicals, and polyphenolic content	[28]
	TPC was 415 mg GAE/g dried extract) with significant antioxidant activity (IC50: 35.4 ± 0.7 µg/mL. The presence of gallic acid (90.8 mg/g), ellagic acid (36 mg/g), caffeic acid (26.07 mg/g), p-coumaric acid (0.26 mg/g), catechin (9.05 mg/g), epicatechin (0.42 mg/g), and quercetin (1.54 mg/g) was discovered using high-performance liquid chromatography. Tannic acid (188.5 mg/g) was also shown to be the most significant phenolic constituent	[37]
Jamun seed and fruit	The greatest TPC in fruit extract was 1462.37 ± 65.80 mg GAE/100 g, TFC were 424.79 ± 41.31 mg/100 g, and anthocyanin content was 5.32 ± 0.31 mg CYE/g, whereas the maximum TPC and TFC in seed extract were 1863.25 ± 70.83 mg GAE/100 g and 953.91 mg/100 g, respectively. The seeds had no anthocyanin. Jamun is a possible source of antioxidants because to these bioactive substances	[38]
Jamun seed and fruit	Phenolic acids, flavonoids, and anthocyanins are antioxidant chemicals	[28]
Jamun leaves	Antidiabetic, polyphenols, flavonoids, terpenoids, saponins, and glycosides	[39]
Jamun leaves extract	Anti-inflammatory, antioxidant, anticancer, and hypoglycemic agents, polyphenols, and flavonoids	[15]
Jamun leaf	Sitosterol, betulinic acid, categoric acid, quercetin, myricetin, methyl gallate, and kaempferol are examples of flavonoids, terpenoids, and phenolics	[30]
Jamun seed	Antioxidant activity and rich source of protein and calcium	[30]
	Flavonoid, antioxidant phytochemicals, and polyphenolic content	[40]
Jamun seed and fruit	Phenolic acids, flavonoids, and anthocyanins are antioxidant chemicals	[29]

**Table 3:** Therapeutic effects of Jamun.

Target Disease	Main Component	Study Objective	Methodology	Result	References
Obesity	Jamun leaf	Obese rats	Take two groups of rats for this experiment. At 60 days, fat rats were fed MSG, while skinny rats were given Jamun extract	In MSG-induced obese mice, Jamun leaf improved peripheral sensitivity to insulin by inducing insulin release from cells, which was associated with better metabolic results	[32]
	Jamun fruit	C57BL/6 mice	For 8 weeks, C57BL/6 mice were given either a normal diet or HFD with or without Jamun fruit extract (JFE; 100 mg/kg/day) via oral gavage	Jamun fruit as a dietary intervention method for obesity and associated metabolic diseases prevention	[46]
	*Senna auriculata*, *P. emblica*., Jamun Skeels	42 Wistar albino rats (6 normal, 32 are diabetic survival)	Every morning, an oral catheter was used to administer the medication for 6 weeks. At the end of the pharmaceutical treatment session, all of the animals fasted overnight. These parameters were measured in urine or blood the next morning	Ethanol extract from three plants causes morphological alteration in the heart and kidney In certain diabetes problems, the combination was proven to be more beneficial	[49]
	Jamblang leaf extract	Wistar rat 18 (divided into 5 groups)	In this study, high-fat and fructose diets are introduced to Wistar rats and also provided Jamun leaf extract to rats for 28 days. Three treatments were introduced, 100, 150, and 200 mg/kg BW	Jamun leaves can inhibit weight gain in the MS model	[50]
*Diabetes mellitis*	Aqueous extract of Jamun + HFD	Diabetic rats induced via streptozotocin	Antihyperglycemic or Antidyslipidemic activity was checked. Aqueous extract of Jamun at concentrations of 100, 200, and 400 mg/kg BW	Jamun showed preventative and curative results on T2DM	[51]
	Jamun fruit and seed extract	Male diabetic rats	For 60 days, male rats were fed ethanol-extracted seed and fruit diets. Insulin levels and serum glucose were observed	Jamun lowers blood glucose levels and aids in insulin control in hyperglycemic mice	[29]
	Jamun seed powder	Streptozotocin are introduced T2DM Rats	CHO and steroids are present in the sample chemical analysis while alkaloids are also present in the herbs sample. Other toxic metals are also present in a sample. Samples are orally introduced to rats for 22 days	Herbal medicine and its constituents Jamun have antidiabetic properties helpful for the control of triglyceride	[41]
	Jamun and alloxan	Alloxan-induced diabetic rats	They were given an ethanolic extract of Jamun seed at doses of 200 mg/kg and 400 mg/kg BW daily for three weeks. The phenolic chemicals in Jamun seed extract are abundant	The phenolic contents in Jamun seed extracted In alloxan-induced diabetic rats, the extract was found to have considerable radical (DPPH) scavenging action and FBS-reducing potential	[40]
	Maghz-e-Jamun (*Eugenia jambolana*)	Human (52 prediabetics)	Prediabetic patients randomized, single-blind, placebo was conducted twice a day orally (capsule) for 12 weeks	It has a suitable source of lowering glycemic in prediabetic patients	[52]
	Ayurveda drugs (Gudmar, Jamun, Nagarmotha, Sudarshan)	Human Diabetic patients (38-year-old male)	Ayurvedic drugs (Jamun) given orally twice a day with lukewarm water and also with units of insulin in T2DM patients	After 12 weeks, it shows a suitable response in diabetic patients who take Ayurveda drugs It improves the HbA1c by 11.1% to 5.6%	[42]
	Jamun Seed	Adult male Wistar albino diabetic rats	For 12 weeks, they fed rats a high-fat diet while inducing streptozotocin (35 mg/kg) as well as introducing Jamun	At treatment of 21 days, results begin to show that It helps to enhance pancreatic function, prevent diabetes, and reduce insulin resistance	[53]
	Jamun skeel	32 Wistar male rats (weight b/w 200 and 300 g)	Jamun was extracted and dried in powder form and given to Wistar male rats for showing hypoglycemic properties of Jamun	Its components had no significant effect on blood glucose regulation However, the phenolic chemicals found in the plant had antioxidant and hypoglycemic properties	[48]
	Jamun skeel, Brassica alba Rabenh, *Trigonella foenum-graecum*, and *Nigella sativa*	In vitro	Extract ethanol from all seeds by using different methods. To see the phytochemical analysis of all seeds, which is the best one	This research found that all of these medicinal seeds, particularly Jamun, have promise as prepared standard functional goods in traditional medicine and can aid with diabetes treatment	[28]
	Jamun + metformin	Diabetic rats	Rats were observed for eight weeks after receiving metformin SC 100, 200 mg/kg, and metformin 90 mg/kg separately	This combination shows an antihyperglycemic impact than either medicine alone When used with conventional antidiabetic drugs, Jamun seed powder can help those with prediabetes or diabetes	[5]
	Jamun leaves	Diabetic rats	Employed an animal model that was fed an HFD before being treated with Streptozotocin injections (dose 1, 50 mg/kg BW and 2, 100 mg/Kg BW). Then orally administrated Jamun into rats	It shows the antihyperglycemic effect of dramatically lowering fasting blood glucose levels	[54]
Diabetes and obesity	Jamun seed extract	Aged male Wistar albino rats (3–4 months)	For 12 weeks, rats were fed a high-fat diet supplemented with streptozotocin. All studies were completed after 21 days of therapy	In the diabetic group, GLUT-4 expression was down-regulated in skeletal muscle, whereas it was up-regulated in adipose tissue It helps to reduce insulin level	[43]
Hypertension	Jamun pulp	20 male restraint stress mice (divided into 4–5 groups)	Each with five rats: normal control, negative control, and two treatment groups of Jamun pulp with dosages of 100 and 200 mg/g BW for 30 min daily for 7 days	An ethanol extract of Jamun pulp can help chronic restraint stress rats by lowering blood pressure and malondialdehyde (MDA) levels	[34]
	Jamun leaves	Male rat 12 weeks old	Wistar rats were taken from their enclosures and injected with Jamun for research purposes	Jamun has excellent potential to deal with hypertension In the future, it is also used as cardioprotective	[55]
Diabetes and hypertension	Jamun leaf	In vitro	Antiglycant activity (BSA/fructose, BSA/methylglyoxal, and arginine/methylglyoxal). Antioxidant activity (DPPH, ORAC, and FRAP), Inhibiting action against α-amylase, β-glucosidase, and lipase	Jamun leaves antiglycation and hypoglycemic actions provide scientific data to support its usage as an antidiabetic agent	[56]
Diabetes and hyperlipidemia	Jamun seed powder	Human (99 patients)	In this study, double-blind, randomized control trials were conducted in hyperglycemic or dyslipidemia patients. Supplemented 10 g Jamun seed powder twice a day before a meal at 90 days	There are no significant changes in weight, neck circumference, hip circumference, BMI and waist circumference But helpful in controlling the glycemic level and dyslipidemia	[45]
	Jamun leaves	Rats	The antiglycant or antioxidant antiglycant action of Jamun ethanolic extracts, and also their inhibitory potential against glucosidase, amylase, and lipase, were studied. Also, the principal Jamun bioactive components in leaves were detected by HPLC-ESIMS/MS analysis	Jamun has pharmacological potential to manage the T2DM and hyperlipidemia by reducing the working of digestive enzyme	[39]
	*Senna auriculata*, *P. emblica*., Jamun, Skeels	42 Wistar albino rats (6 normal, 32 are diabetic survival)	Every morning, an oral catheter was used to administer the medication for 6 weeks. At the end of the pharmaceutical treatment session, all of the animals fasted overnight. These parameters were measured in urine or blood the next morning	Ethanol extract from three plants causes morphological alteration in the heart and kidney In certain diabetes problems, the combination was proven to be more beneficial	[49]
Hyperlipidaemia	Jamun seed	Human (male and female at age of 18–65 years)	A pilot study was conducted on prediabetic patients; patients were split up into two groups, one of which introduced powder of Jamun seed at 4.5 g/day, and 2nd group was given placebo capsules for 84 days The patient was screened in OPDS IPDS	Jamun was found to play a significant influence in decreasing lipid profiles in people with prediabetes	[21]
	Jamun seed and leaves	20 male hyperlipidemic rats	The antihyperlipidemic activity was checked in albino rats. These rats were separated into four groups, each with its feeding regimen. Following the experiment, a lab test was performed for analysis	Jamun seeds and leaves are both effective in the treatment of hyperlipidemia. Also, improve the function of the liver	[30]
	Jamun skeel freeze-dried fruit flesh	Hyperlipidemic mice	In this facade study, Jamun skeel freeze-dried fruit flesh is introduced to five groups of mice for 9 days	It helps to increase the HDL level and also shows the beneficial effect of TAG It did not produce anti-obesity effects	[8]
Antioxidative and anti-inflammatory	Jamun leaves extract	Rabbits (6–8) and rats (2)	Jamun leaf extract was introduced in rabbits and rats (at 22 °C)	Jamun leaves contain antioxidant and anti-inflammatory effects. They can be used to treat cardiovascular illnesses and malignancies	[15]
	Jamun skeels fruit extracts	Wistar albino rats and mice	Using the HPLC method for the determination of active compounds in Jamun and the introduction to the rat that shows anti-inflammatory properties	Fruit extracts from Jamun are a plentiful source of bioactive substances that have anti-inflammatory properties	[47]

## Data Availability

Not applicable.
